# Seroepidemiology and molecular diversity of *Leishmania donovani* complex in Georgia

**DOI:** 10.1186/s13071-016-1558-6

**Published:** 2016-05-13

**Authors:** Giorgi Babuadze, Jason Farlow, Harry P. de Koning, Eugenia Carrillo, Giorgi Chakhunashvili, Mari Murskvaladze, Merab Kekelidze, Irakli Karseladze, Nora Kokaia, Irine Kalandadze, David Tsereteli, Ivane Markhvashvili, Ketevan Sidamonidze, Gvantsa Chanturia, Ekaterine Adeishvili, Paata Imnadze

**Affiliations:** National Center for Disease Control and Public Health of Georgia, 9 M. Asatiani Str. 0186, Tbilisi, Georgia; Ilia State University, Tbilisi, Georgia; Farlow Scientific Consulting Company, Lewiston, UT USA; Institute of Infection, Immunity and Inflammation, College of Medical, Veterinary and Life Sciences, University of Glasgow, Glasgow, G43 2DX UK; Unit of Leishmaniasis and Chagas Disease, WHO Collaborating Centre for Leishmaniasis, National Center for Microbiology, Institute of Health Carlos III, Madrid, Spain; S. Virsaladze Research Institute of Medical Parasitology and Tropical Medicine, Tbilisi, Georgia

**Keywords:** Epidemiology, Visceral leishmaniasis, Phylogeny of *Leishmania*, ITS Sequencing, Georgia, *Phlebotomus*

## Abstract

**Background:**

Leishmaniasis includes multiple clinical syndromes, most notably visceral, cutaneous, and mucosal forms. Visceral leishmaniasis (VL), also known as kala-azar, is a potentially fatal disease endemic to large parts of Africa and Asia, and in South-Eastern Europe (Greece, Turkey, Georgia). Visceral leishmaniasis is a parasitic zoonosis caused by species of the *L. donovani* complex. In the classical epidemiological model the main reservoir for VL are canines.

**Methods:**

The study included a cohort of 513 individuals of both genders (190 males and 323 females) from the ages of 1 to 70 years that were screened in ten villages across two districts in Kakheti using the Kalazar Detect™ rK39 rapid diagnostic test. The phylogenetic diversity patterns of local strains, based on the rDNA internal transcribed spacer (ITS) sequences, were assessed for samples obtained from patients with suspected *L. donovani* infection, from canine reservoirs and from *Phlebotomus* sand flies obtained from different geographical areas of Georgia and from Azerbaijan.

**Results:**

Out of a total of 600 domestic dog blood samples 95 (15.8 %) were positive by rK39 rapid diagnostic tests. For symptomatic domestic dogs, the testing of conjunctival swabs or bone marrow aspirates revealed a higher VL incidence in Kvareli District (Kvareli; 19.4 %, *n* = 329) compared with that observed for Sagarejo District (Sagarejo; 11.4 %, *n* = 271). A total of 231 sand flies of both genders were collected during the 2-month period; of the 114 females, 1.75 % were PCR positive for the presence of *Leishmania* spp.

**Conclusions:**

VL infection rates remain high in both canines and humans in Georgia, with disease in several known natural foci. The genetic relationships derived from rDNA internal transcribed spacer (ITS) sequence comparisons identified genetic subgroups, revealing preliminary insights into the genetic structure of *L. donovani* complex members currently circulating in the South Caucasus and demonstrates the utility of ITS-based genotyping in the resource-limited country of Georgia.

**Electronic supplementary material:**

The online version of this article (doi:10.1186/s13071-016-1558-6) contains supplementary material, which is available to authorized users.

## Background

Leishmaniasis is a complex of diseases caused by intracellular parasitic protozoans of the genus *Leishmania*, a representative of the order Kinetoplastida, family Trypanosomatidae. The two most common manifestations of the disease are the cutaneous form, which is largely self-healing but often leaves disfiguring scars, and the usually fatal visceral form. Estimated cases of visceral leishmaniasis (VL) have reached 300,000 globally [1], with a high rate of HIV co-infections, and over 20,000–40,000 deaths annually [2, 3]. In addition, a high incidence of canine viscerocutaneous leishmaniasis, as well as an increasing burden of human VL, is observed throughout European Mediterranean and East European countries [2, 4] and is spreading into central Europe [5, 6]. The genus *Leishmania* is currently divided into several species complexes. Numerous *Leishmania* species have been reported in Eurasia and Africa, including *L. infantum, L. major, L. donovani, L. tropica, L. aethiopica, L. turanica, L. gerbilli, * and *L. arabica* [7]*. L. infantum*/*chagasi* and *L. donovani* cause VL with some minor differences, for which various species of canids, in particular, serve as the animal reservoir, although in India/Nepal/Bangladesh VL is considered as principally antroponotic [2].

**Fig. 1:**
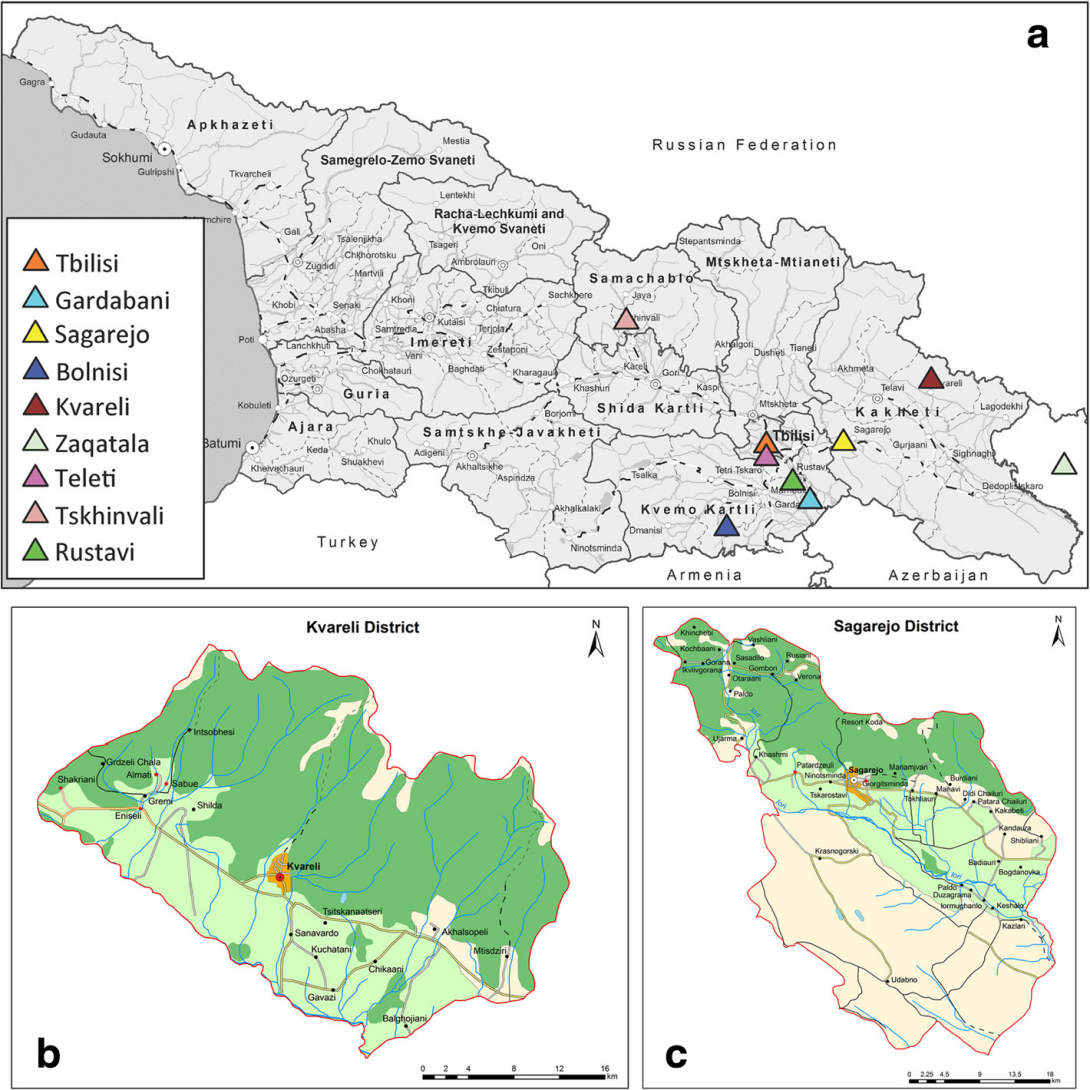
Map of Georgia and detailed maps of the study areas. **a** Map of Georgia. **b** Map of Kvareli District. **c** Map of Sagarejo District. The red spots indicate the villages where rk39 test- positives were found

VL presents one of the most serious public health concerns in Georgia [8, 9]. The first known outbreak of VL in Georgia, in 1913, occurred in the eastern-most part of the country (Kakheti region) and gave rise to the first clinical report of this disease in the Caucasus region. In 1954 a survey found 540 cases in eastern Georgia [10]: cases were registered in six cities and 164 villages, mainly in the Kakheti region in the east of the country, but also in the more central region of Shida Kartli [11] (see map in Fig. 1a). In the 1960s large scale malaria control efforts were carried out in eastern Georgia using massive spraying campaigns with dichlorodiphenyltrichloroethane (DDT) [11], which significantly reduced the local sand fly population in addition to the intended targets, the *Anopheles* mosquitoes spreading malaria. This historical spraying campaign is now generally considered to be the primary cause of the reduction in reported VL cases over the subsequent 40 years. As a consequence, however, there has been an almost complete lack of biosurveillance in eastern Georgia for four decades, and therefore there are no reliable data on clinical and sub-clinical prevalence of leishmaniasis in the region for this period.

Historically, isoenzyme analysis has been the gold standard for *Leishmania* species and strain identification and discrimination. Due to the propensity for artefactual outcomes derived from this method, molecular assays have largely replaced isoenzyme-based assays for this purpose. Several molecular typing techniques have shown utility in distinguishing individual species of *Leishmania*, including random amplification of polymorphic DNA (RAPD) [12], amplified fragment length polymorphism (AFLP) [13, 14], kinetoplastid DNA (kDNA) PCR-based restriction fragment length polymorphism (RFLP) [15], microsatellite sequencing [12], and SCAR (sequence-confirmed amplified region) analysis [16]. In addition, ribosomal internal transcribed spacer (ITS) sequences have previously characterized genetic relationships within the *Leishmania*, providing key information about leishmaniasis distribution and population structure [12–14].

We report here the seroepidemiology and genotyping of VL foci in the eastern half of Georgia. Limited resources are available in this country and we present the first report of *Leishmania* strain detection and genotyping in Georgia. Phylogenetic analysis of ITS-based Georgian VL samples, collected over a 2-year period, revealed preliminary insights into the genetic structure of *L. donovani* complex members currently circulating in Georgia. This has identified new genotypes that are unique to Georgia, has provided much-needed insights into the spread of leishmaniasis in the south Caucasus region, and informs rational intervention strategies needed to curb the resurgent VL epidemic in the region. In addition, it demonstrates the utility of ITS-based genotyping in resource-limited settings.

## Methods

In order to obtain data on VL prevalence and disease burden within the human population and the canine reservoir, seroepidemiological surveys were performed in two historically active VL foci in the Kakheti region: Kvareli and Sagarejo districts (Fig. 1b and c). Infection screening was performed on domestic dogs in the selected districts, using the Kalazar Detect™ rK39 rapid diagnostic test (rK39). Collection and taxonomic identification of sand fly species as potential *Leishmania* vectors was performed and the presence of *Leishmania* organisms was confirmed using microscopy and PCR. Amplification and sequencing of the ITS region of 19 isolates allowed the molecular genotyping necessary to identify the geographical spread of unique strains, and their comparisons with strains that have previously been reported from other regions.

### Study area

Georgia is located on the crossroads of Europe and Asia. It lays at the eastern end of the Black Sea, with Turkey and Armenia to the south, Azerbaijan to the east and Russia to the north, across the Caucasus Mountains. The capital and largest city is Tbilisi. Georgia’s total territory covers 69,700 km^2^. Georgia is divided into nine regions and two autonomous republics (Fig. 1a). These, in turn, are subdivided into 69 districts.

Kakheti is a region in eastern Georgia. The region comprises of eight administrative districts: Telavi, Gurjaani, Kvareli, Sagarejo, Dedoplistsqaro, Signagi, Lagodekhi and Akhmeta. Kakheti is bordered by the Russian Federation to the north-east, Azerbaijan to the south-east, and the Georgian regions of Mtskheta-Mtianeti and Kvemo Kartli to the west (Fig. 1a). Two of the above listed districts of Kakheti region were chosen for this study: Kvareli and Sagarejo. Our attention was drawn to these areas in particular because of a significantly increased number of VL cases since 2009, up from at most sporadic registered cases to 4–9 clinical cases in each district annually, making those two districts among the most affected with VL infection in the Kakheti region [official disease records, National Centre for Disease Control (NCDC), Tbilisi, Georgia].

Kvareli District is a municipal district and administrative-territorial unit in East Georgia, of mostly agricultural lands and forest, 133 km from Tbilisi, in the north-eastern part of the Kakheti region at 41.9514 °N, 45.8017 °E. The terrain of Kvareli district (1001 km^2^) is diverse and lies at an elevation of 255–1800 m, with 40,200 inhabitants (census 2012), near the foothills of the Greater Caucasus Mountains. Sagarejo District is situated 58 km to the east of Tbilisi, and has a population of 59,212 (2002 census). The district is situated at an elevation of 460–1800 m, with an area of 1491 km^2^, containing forests, vineyards and a large segment of desert-like terrain. Apart from the desert part of Sagarejo District, the climate of the two study areas is very similar, with hot summers creating the conditions for breeding of *Phlebotomus* sand flies.

### Ethics statement

Ethical clearance for conducting this study was granted by the Institutional Board of Review of the National Center for Disease Control and Public Health (IRB00002150) in compliance with Georgian legislation and international bioethical frameworks. All volunteers were interviewed and written informed consent was obtained for participation in the study. Under the current state regulations no special permission was required for the research and diagnostic procedures utilized for the pet animal work in this study. However written informed consent was obtained from the particular pet owners.

Investigators and animal care staff had appropriate qualifications and experience for conducting the procedures described below on living animals. Each animal received compassionate care, comfort, and protection from abuse and unnecessary pain. In order to obtain the bone marrow aspirates, a mixture of 2 % Xylazine (1–2 mg/kg) and 5 % Kaipsol (5–10 mg/kg) was used for anesthesia and myorelaxation. Investigators minimized distress in individual animals or populations of animals in accordance with the European Convention for the Protection of Vertebrate Animals Used for Experimental and Other Scientific Purposes, Strasbourg 1986.

### Study design and sampling

#### A. Human subjects

For the selection of human subjects, a survey was performed in households in the district, starting with households from which a clinical case of VL had been reported, preferentially a person aged 0–14 (‘new’ cases as opposed to historical cases). Among 175 households, 513 human subjects (190 males and 323 females) from 1 to 70 years (average = 24.2; median = 23) were enrolled from ten villages of the two Kakheti districts. Serum samples were obtained from all 513 individuals over a 2-month period from June to July 2014. One of the objectives of the study was to determine the transmission risk-factors in the Kakheti region districts where confirmed VL cases have increased in the last ten years. For this purpose, we included the following criteria for the enrolment of study subjects: (i) human subjects were a member of a household where confirmed cases were registered since 2009; or (ii) subjects would reside close to a previously reported case. Additionally, it was decided that no more than three human samples (and a maximum of five samples including dogs and vectors) were to be collected per household in order to avoid sample bias. In both districts, virtually all (> 95 %) households have at least one dog, especially in the outlying villages. Human blood samples were collected in Vacutainer vials designed for serum and stored at 4 °C. During this survey a total of seven rK39 test-positive persons were identified, who were referred to the Institute of Medical Parasitology and Tropical Medicine in Tbilisi, for confirmatory tests on bone marrow aspirates. PCR testing of bone marrow samples, provided by the Institute of Medical Parasitology and Tropical Medicine, was performed at the R. Lugar Center, Tbilisi.

#### B. Canine and vector sampling

A total of 600 asymptomatic dog samples (blood and serum) were obtained during the three month period June–August, 2014 in both districts (*n* = 329, Kvareli District and *n* = 271, Sagarejo District). Dog sampling was implemented in the same settlements where human subject and vector survey samples were collected. Canine blood samples were stored and tested under similar conditions as the human samples. Vector surveys were simultaneously implemented in Kvareli and Sagarejo districts (July–August, 2014), using seven standard CDC light traps [17]. For each VL focus the light traps were set between 7 pm and 7 am on four consecutive nights per week. Sand fly species was determined according to the morphological ID keys and scored according to blood fed versus unfed [18]. A total of 114 of the 231 sand flies collected were female (*n* = 162, Kvareli and *n* = 69, Sagarejo District). For detection of *Leishmania* parasites all female sand flies were placed in 1× sterile PBS buffer and stored at 4 °C for further PCR testing.

### rK39 testing

For the detection of VL antibodies in human and dog serum samples Kalazar Detect™ rK39 Rapid tests (InBios International Inc, Seattle, USA) were performed according to the manufacturer’s instructions. A total of 20 ml was collected for each human serum sample. The test was assumed positive when a control line and test line appeared on the dipstick within 10 min, and negative if only a single control line appeared [19, 20].

### Culture

Culture tubes containing Novy-MacNeal-Nicolle (NNN medium) agar with 10 % defibrinated rabbit blood were overlaid with 200 μl of complete RPMI medium containing 20 % fetal bovine serum. Several drops of human bone marrow and canine spleen aspirates (collected in EDTA tubes from Becton, Dickinson and Company, USA) were introduced into the tubes containing NNN agar and RPMI medium. For sand fly samples, mid guts were extracted and diluted in two drops of sterile PBS buffer, which was finally transferred to NNN medium with a 2-ml syringe. The tubes were incubated at 27 °C; and were examined by light microscopy two times per week until promastigotes were noted or several weeks had passed. The two cultures thus obtained (one from a canine spleen aspirate and another from a phlebotomine sand fly) were transferred from tubes to small flasks containing 10 ml of fresh RPMI medium in order to obtain large numbers of parasites for further investigations. Cultures were passaged several times. Once the parasites reached log phase, liquid culture medium was harvested from the flasks and centrifuged at 1000 × g for 10 min. Supernatants were discarded and pellets washed in 50 ml of 1 % Hank’s + D-Glucose medium (pH 7.2) prior to DNA extraction.

### DNA extraction and PCR amplification

DNA was extracted using the QIAamp DNA Mini Kit (Qiagen) according to the manufacturer’s instructions. A total of 19 isolates were used for phylogenetic analysis in this study. One of the isolates obtained was from a patient from the Azerbaijani village Zakatala, who had been referred to the Institute of Medical Parasitology, Tbilisi, Georgia, for confirmatory test and further treatment.

Sources, designation and geographical origins of the samples used in this study are listed in Tables 1 and 2. For the initial evaluations of the *Leishmania* ITS region, the 1030 bp ribosomal ITS region was amplified and sequenced using *Leishmania*-specific primers: LITSR (5′ - CTG GAT CAT TTT CCG ATG - 3′) and LITSV (5′- ACA CTC AGG TCG TAA AC - 3′) [14]. Separately were amplified ITS1 - LITSR (5′- CTG GAT CAT TTT CCG ATG - 3′)/L5.8S (5′ - TGA TAC CAC TTA TCG CAC TT- 3′), ITS2 - L5.8SR (5′ - AAG TGC GAT AAG TGG TA - 3′)/LITSV (5′- ACA CTC AGG TCG TAA AC - 3′) [14] and ITS2 generic primers - LGITS2F2 (5′- GCA TGC CAT ATT CTC AGT GTC - 3′)/LGITS2R2 (5′ - GGC CAA CGC GAA GTT GAA TTC - 3′) [21].

**Table 1:** Sample description of Georgian sequences used in this study

Origin	Type	Geographical location	Year	ITS sequence type	Accession number	Clinical presentation
Human	DNA	Gardabani	2013	ITS1/2	MM201501	Yes
Human	DNA	Tkhinvali	2014	ITS1/2	MM201502	Yes
Human	DNA	Bolnisi	2014	ITS1/2	MM201503	Yes
Canine	DNA	Kvareli	2014	ITS1/2	MM201504	no
Canine	DNA	Sagarejo	2014	ITS1/2	MM201505	No
Canine	DNA	Sagarejo	2014	ITS1/2	MM201506	No
Human	DNA	Rustavi	2014	ITS1/2	MM201507	yes
Canine	DNA	Sagarejo	2014	ITS1/2	MM201508	Yes
Canine	Culture	Tbilisi	2014	ITS1/2	MM201509	Yes
Canine	DNA	Tbilisi	2012	ITS1/2	MM201511	No
Canine	DNA	Tbilisi	2012	ITS1/2	MM201512	No
Canine	DNA	Tbilisi	2012	ITS1/2	MM201513	No
Canine	DNA	Tbilisi	2012	ITS1/2	MM201514	No
Human	DNA	Tbilisi	2012	ITS1/2	MM201515	No
*P. balcanicus*	DNA	Tbilisi	2012	ITS1/2	MM201516	
*P. balcanicus*	DNA	Tbilisi	2012	ITS1/2	MM201517	
Human	DNA	Zakatala	2014	ITS1/2	MM201519	Yes
Canine	DNA	Tbilisi	2013	ITS1/2	MM2015120	No
Canine	DNA	Teleti	2013	ITS1/2	MM2015121	No

**Table 2:** Sample description used for phylogenetic analysis in this study

WHO number	Origin	ITS sequence type	Accession number
MHOM/BR/74/PP75	Brazil	ITS	AJ000304
	Brazil	ITS2	GU045591
MHOM/IN/71/LRC-L51a	India	ITS1	AJ000290
MHOM/SD/75/LV139	Sudan	ITS	AJ000291
MHOM/IN/80/DD8	India	ITS	AJ000292
MHOM/SD/68/1S	Sudan	ITS	AJ000293
MHOM/KE/84/NLB218	Kenya	ITS	AJ000296
MHOM/KE/85/NLB323	Kenya	ITS	AJ000297
MCAN/SD/00/LEM3946	Sudan	ITS1/2	AJ634356
MHOM/SD/93/GE	Sudan	ITS1/2	AJ634357
MHOM/SD/97/LEM3429	Sudan	ITS1/2	AJ634358
MHOM/ET/00/HUSSEN	Ethiopia	ITS1/2	AJ634360
MHOM/SD/62/LRC-L61	Sudan	ITS1/2	AJ634365
MHOM/SD/93/35-band	Sudan	ITS1/2	AJ634366
MHOM/ET/72/GEBRE1	Ethiopia	ITS1/2	AJ634367
MHOM/SD/93/338	Sudan	ITS1/2	AJ634368
MHOM/SD/93/9S	Sudan	ITS1/2	AJ634372
MHOM/ET/67/HU3	Ethiopia	ITS1/2	AJ634373
MHOM/KE/83/NLB189	Kenya	ITS1/2	AJ634374
MHOM/IN/54/SC23	India	ITS1/2	AJ634375
MHOM/IN/00/DEVI	India	ITS1/2	AJ634376
MHOM/IN/96/THAK35	India	ITS1/2	AJ634377
MHOM/IN/01/BHU20140	India	ITS1/2	AJ634378
		ITS1/2	FJ753386
		ITS1/2	GU045589
		ITS1/2	GU045590
MHOM/CN/00/Wangjie1	China	ITS	AJ000294
MHOM/SU/1984/MARZ/KRIM	Ukrain	ITS	AM157172
MHOM/IQ/1981/SUKKAR2	Iran	ITS1	AM901452
MHOM/FR/78/LEM75	France	ITS1/2	AJ634339
MHOM/FR/95/LPN114	France	ITS1/2	AJ634340
MHOM/ES/93/PM1	Spain	ITS1/2	AJ634341
MHOM/FR/97/LSL29	France	ITS1/2	AJ634342
MHOM/ES/86/BCN16	Spain	ITS1/2	AJ634343
MHOM/PT/00/IMT260	Portugal	ITS1/2	AJ634344
MHOM/CN/54/Peking	China	ITS1/2	AJ634345
MCAN/FR/87/RM1	France	ITS1/2	AJ634346
MHOM/ES/88/LLM175	Spain	ITS1/2	AJ634347
MHOM/MT/85/BUCK	Malta	ITS1/2	AJ634350
MHOM/FR/80/LEM189	France	ITS1/2	AJ634351
MHOM/ES/92/LLM373	Spain	ITS1/2	AJ634352
MHOM/IT/94/ISS1036	Italy	ITS1/2	AJ634353
MHOM/IT/93/ISS800	Italy	ITS1/2	AJ634354
MHOM/SD/93/597-2	Sudan	ITS1/2	AJ634364
MHOM/SD/82/GILANI	Sudan	ITS1/2	AJ634369
MHOM/SD/93/452BM	Sudan	ITS1/2	AJ634371
MHOM/TN/80/IPT1	Tunisia	ITS	AJ000289
	India	ITS1/2	FJ948458
MHOM/ET/72/L100	Ethiopia	ITS1/2	GQ920674

For the PCR amplification, 3–10 ng of DNA was added into a mix containing 10× reaction buffer, 0.2 mM dNTP’s, 0.2 μM of each primer, nuclease-free distilled water (Sigma-Aldrich) and 2 units of *Taq* polymerase (Biotools). PCR was performed in a thermocycler TC-5000 (TECHNE), using the following cycle parameters: 94 °C for 2 min, followed by 40 cycles of 94 °C for 30 s, 55 °C for 30 s, 70 °C for 1 min, and a final extension of 72 °C for 10 min. The amplicons were purified by precipitation, first with ice-cold 100 % and then 70 % 2-Propanol. Sequencing reactions of the amplicons were carried out with the BigDye Terminator Kit, version 3.1 (Applied Biosystems). For further steps sequencing reaction mixtures were purified by again washing with 100 % and 70 % 2-Propanol. The purified product was transferred into Applied Biosystems ABI PRISM 96-Well optical reaction plates; to each well 10 μl Hi-Di formamide (Thermo Fisher) was added. Subsequently products were denatured at 94 °C for 2 min in a thermal cycler and finally analyzed on an ABI Prism 3130 XL sequencer, with data collection software version 3.0.

### Sequencing by Sanger method and phylogenetic analysis

Samples were considered PCR positive if a PCR product of approximately 1030 bp for the whole ITS region was obtained; ITS, ITS1, ITS2 and ITS2 (generic primers) were all amplified separately. PCR fragments were sequenced in both directions to assure sequence accuracy. DNA sequences were edited using the Sequencher® sequence analysis software (version 5.3; Gene Codes Corporation, Ann Arbor, MI, USA) and deposited in GenBank under accession numbers KT438661–KT438681.

The 19 Georgian rDNA ITS1 and IT2 sequences were concatenated and manually aligned using MEGA v6 software [22]. ITS1 and ITS2 region boundaries for all sequences were derived from primer-delimited sequences. A total of 51 sequences included from BLAST searches against the NCBI nucleotide database were chosen based on high similarity score. Genetic distances between rDNA sequences were estimated using the MEGA v6 software [22]. The best fit model was determined using the ML method based on Akaike Information Criterion (AIC) and Bayesian Information Criterion (BIC) values implemented in MEGA v6 [22]. The Jukes-Cantor (JC) model [23] was recommended by both AIC and BIC. Non-uniformity of evolutionary rates among sites was modeled by using a discrete Gamma distribution (+G). Phylogenetic analysis based on the nucleotide sequences of concatenated *Leishmania* ITS1 and ITS2 sequences was performed using Neighbor joining (NJ) and Maximum Likelihood (ML) algorithms performed in MEGA v6 software. The rate variation among sites was modelled with a gamma distribution (shape parameter = 1) with partial gap deletion. The analysis involved 68 nucleotide sequences. The bootstrap method was used to assess branch support (1000 replicates).

### Statistical data analysis

Data analysis was performed by using SPSS software version 20 (IBM), which was used to determine the statistical significance of differences between the groups by using Pearson’s chi-squared test. A *P* value of <0.05 was considered statistically significant.

## Results

### Prevalence of leishmaniasis in the human populations of Kakheti region

Seven individuals aged 3 to 56 years old were positive in the rK39 tests (average age 22.7, median age 9 years old). The majority of the human subjects (5/7) resided in Kvareli district. Of the seven patients, six were newly confirmed cases and one (aged 56) had previously been treated for relapse after treatment with liposomal amphotericin B (AmBisome®). Four of the six newly confirmed cases were asymptomatic while two displayed mild symptoms including fever and anemia. Subsequently, all six patients underwent a full course of treatment with an increased dose of AmBisome at the Institute of Medical Parasitology and Tropical Medicine, Tbilisi, making a full clinical recovery.

### Infection in canines

Seropositive dogs were found in every settlement of both foci enrolled in the study. The test results shows that the prevalence of seropositive dogs in Kvareli District is significantly higher (19.5 %; 64/329) 272 than in Sagarejo district (11.4 %; 31/271) (*χ*^*2*^ = 7.301, *df* = 1, *P* = 0.0069). The highest rates of canine seropositivity were 37.3 % (19/51) and 36.4 % (20/55), detected in two villages of Kvareli District, Almati and Sabue, which are located within 2 km of each other (Fig. 1a). Dogs were grouped by their size (large, medium, and small breeds) and by age (≤ 1.5 years; 1.5–5 years; ≥ 5 years old). There was no significant correlation between seropositivity and either the size of breed (Table 3) or sex of the dogs (*χ*^*2*^ = 2.337, *df* = 1, *P* = 0.126). However, seroprevalence was significantly higher in older dogs ≥ 1.5 compared to dogs under 1.5 years of age (*χ*^*2*^ = 19.368, *df* = 1, *P* < 0.0001); interestingly, seroprevalence did not further increase in dogs > 5 years age compared to those between 1.5 and 5 years old (Table 4) (*χ*^*2*^ = 0.049, *df* = 1, *P* = 0.8256) (Table 4).

**Table 3:** VL seroprevalence in dogs categorized by breed and age

Breeds	Small				Medium				Large				
Age	≤ 1.5	1.5–5	> 5	Total	≤ 1.5	1.5–5	> 5	Total	≤ 1.5	1.5–5	> 5	Total	Grand total
Total	82	104	48	234	76	121	48	245	46	56	19	121	600
Positive	8	18	9	35	3	30	12	45	2	9	4	15	95
(%)	9.8	17.3	18.8	15	3.9	24.8	25.0	19	4.3	16.1	21.1	13	15.8

No statistical association was found between seroprevalence and size of breed VL (*χ* ^2^ = 2.337, *df* = 1, *P* = 0.126)

**Table 4:** VL seroprevalence in dogs of the different age categories

Age (years)	≤ 1.5	1.5–5	> 5
Total dogs	204	281	115
Positive	13	57	25
Negative	191	224	90
Percent	7.0	21	22
Chi square test (relative to age ≤ 1.5)		*χ* ^2^ = 18.022, *df* = 1, *P* < 0.0001	*χ* ^2^ = 15.178, *df* = 1, *P* < 0.0001

Data shown are appended from Table 1, showing totals for age of all three size categories. There was no significant difference in seroprevalence between age categories 1.5–5 and >5 (*χ* ^2^ = 0.049, *df* = 1, *P* = 0.8256)

### Identification of *Leishmania* vectors

To expand our previous surveys in Tbilisi and Kutaisi [8] we collected specimens of *Phlebotomus* in Kvareli and Sagarejo districts. In Kvareli we identified *P. balcanicus, P. kandelakii* and *P. sergenti * but only two of these species were found to be present in Sagarejo District (*P. balcanicus* and *P. kandelakii*). All collected female sand flies were tested for *Leishmania* parasites by nested polymerase chain reaction (Ln-PCR). Two *Phlebotomus* flies, both *P. balcanicus* from Kvareli District, were PCR positive: 2.6 % out of 78 female sand flies collected in the district (Table 5). No infected sand flies were found in the sample collected in Sagarejo District. In Kvareli District the most abundant species was *P. balcanicus* (29/78); in Sagarejo District *P. kandelakii* was the most abundant (27/39; Table 5).

**Table 5:** *Phlebotomus* species identified in Kvareli District and Sagarejo District

Species	Kvareli District				Sagarejo District			
	Male	Female	Positive	% of female	Male	Female	Positive	% of female
*P.* (*Adlerius*) *balcanicus*	24	29	2	6.9	10	9	0	0.0
*P.* (*Paraphlebotomus*) *sergenti*	14	22	0	0	0	0	0	0.0
*P.* (*Larroussius*) *kandelakii*	46	27	0	0	23	27	0	0.0
Total	84	78	2	2.6	33	36	0	0.0

Each female sand fly was tested and examined for *Leishmania* parasites microscopically and by PCR; both the number and percentage of *Leishmania*-positive flies are listed

### Genetic relationships

**Fig. 2:**
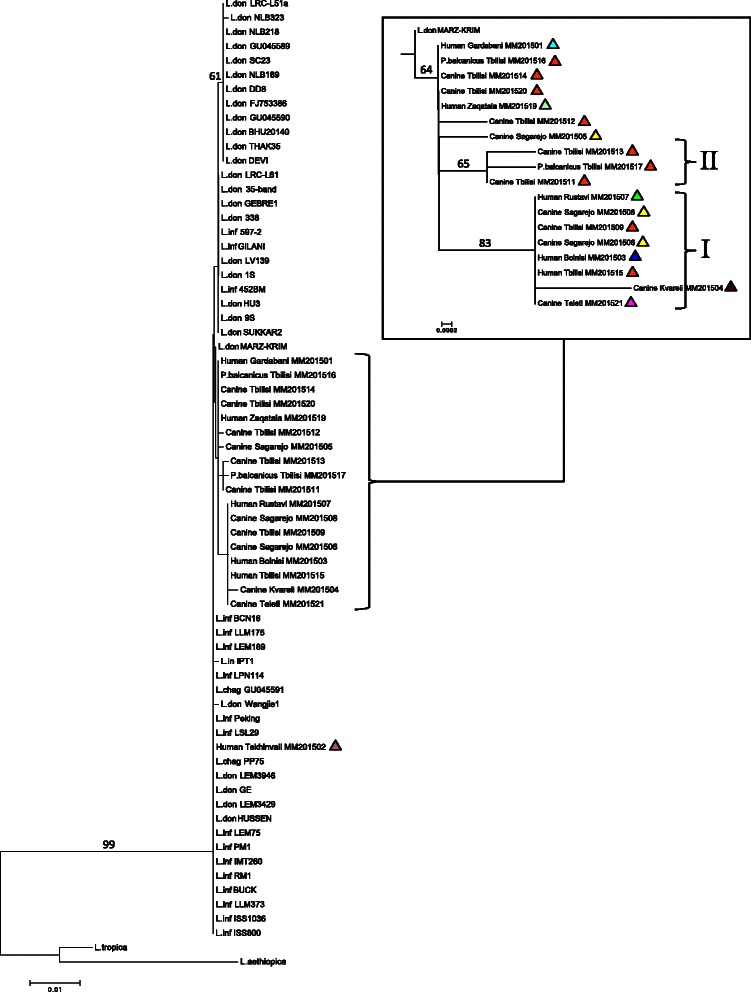
Phylogenetic relationships among concatenated *L. donovani* complex rDNA ITS sequences in this study. The consensus neighbor-joining dendogram was inferred from maximum likelihood (ML) analysis of concatenated ITS1/ITS2 sequences under the JC best-fit model. *Colored triangles* adjacent to each strain identifiers in the dendogram refer to geographical locations listed in Fig. 1

Phylogenetic analysis of the ITS1/2 sequences derived from Georgian isolates showed close genetic association with the ITS1/2 sequences of representative isolates within the *L. donovani* complex. A single monophyletic subgroup was observed that comprised a distinct set of isolates (Group I). NJ and ML trees resolved eight of the 19 Georgian isolates into a single monophyletic subgroup, arbitrarily designated I, with significant bootstrap support (83 and 86 %, respectively) (Fig. 2, Additional file 1: Figure S1). A second group (II) was comprised of three isolates associated together in all trees analyzed albeit with non-significant bootstrap support (65 and 61 %, respectively, for the NJ and ML trees) (Fig. 2, Additional file 1: Figure S1). Both genetic groups showed alternate topological placement respective to alternate phylogenetic algorithms. Although high bootstrap support was not obtained for the Group II Georgian isolates, they alone share an A28G variation in the poly-A tract of ITS1 not found in any of the other Georgian isolates, or in fact in any *Leishmania* strain sequenced to date (Additional file 2: Figure S2). In addition, these three isolates are distinguished from other Georgian isolates by sharing a single A insertion at position 34 that is also present in some *L. infantum* and *L. donovani* strains from other geographical locations.

NJ analysis distinguishes a single assemblage for all Georgian isolates with the exception of the human isolate from Tskhinvali, although bootstrap support was not significant under the best fit evolutionary model used (Fig. 2). This sample was isolated from a 15-month old male patient who had never traveled outside his district, demonstrating local transmission of the strain. In contrast, ML analysis indicated a paraphyletic relationship among Georgian isolates that includes the monophyletic subgroup arbitrarily designated here as Group I, which was also robustly supported in the NJ tree topology (Additional file 1: Figure S1). Group I is comprised of a total of eight human and canine isolates including representatives from Tbilisi, Sagarejo, Rustavi, Kvareli, Bolnisi and Teleti (BS = 81), showing that this genotype is geographically widespread in Eastern Georgia and may have a shared common ancestor. The three genetically similar (but unique compared to all other isolates) strains of Group 2 had all been isolated in Tbilisi (Group II) and this genotype has not been observed elsewhere in Georgia.

We noted seven arbitrary diversity regions (DR) designated DR1-7 among the combined ITS sequences analyzed (Additional file 2: Figure S2). Nucleotide variations comprised single nucleotide polymorphisms (SNPs), insertion/deletions (InDels) and microsatellite copy number variations that are consistent with those previously reported for the *donovani/infantum* complex (Additional file 2: Figure S2, Additional file 3 - alignment file).

## Discussion

**Fig. 3:**
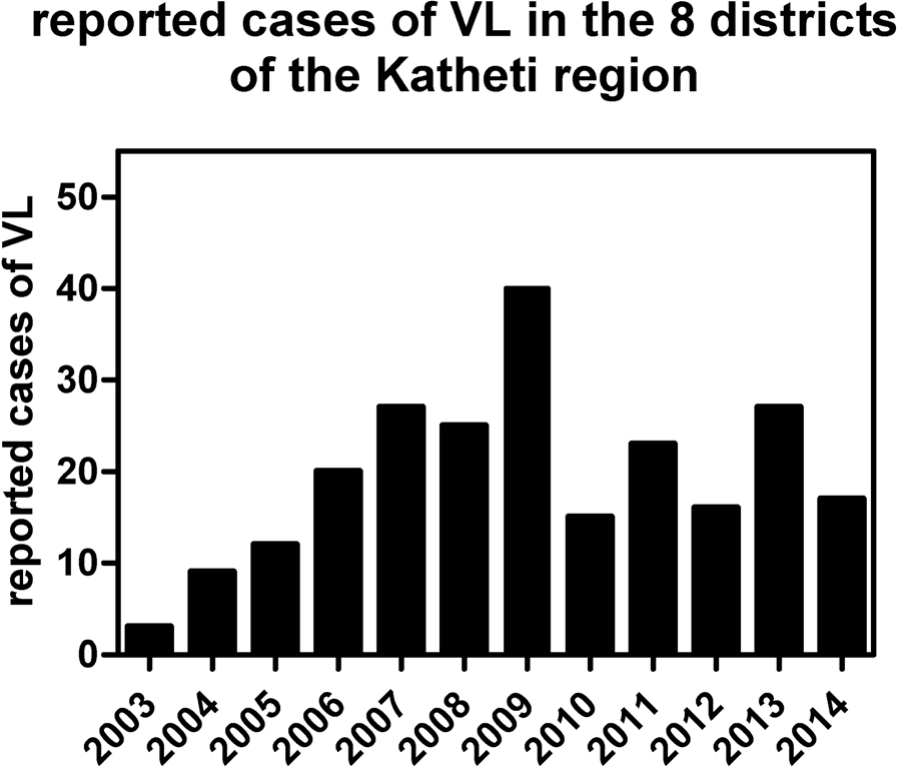
Dynamics of VL cases during the 12 years (from 2003 up to 2014) by years in Kakheti region

We have recently published a survey of *Leishmania* infection in Tbilisi and in Kutaisi, a fairly large city in Central Georgia [8]. The study addressed infection rates in *Phlebotomus* sand flies, canines and humans, and found particularly high incidence in the capital, but also transmission by infected vectors in Kutaisi. We tentatively concluded that leishmaniasis was spreading westward in the country, as the disease had not previously been reported as far west as the Kutaisi area, although it was noted that several different subspecies of *Phlebotomus* were identified compared to the capital. Historically, however, leishmaniasis has been much more prevalent in the east of the country, including Tbilisi. Indeed, there has been a recent surge in reported cases from the Kakheti region, east of the capital, which borders Azerbaijan and the Russian Federation. Between 2007 and 2014, 190 clinical cases of VL were reported in the Kakheti region, compared to just three in 2003 and nine in 2004 (Fig. 3). We thus decided to investigate two Kakhetian districts, Kvareli and Sagarejo, that reported relatively high incidence, of 21 and 26 cases, respectively, in 2007–2012 (41 % of reported cases in Kakheti); previously only sporadic cases (less than one per year) had been reported from these areas. It must be stressed that these statistics reflect purely passive case-finding, i.e. patients presenting themselves with clinical symptoms, and correctly diagnosed with VL. Here, we report an active survey of human, canine and phlebotomine hosts in these districts to identify infections in the active foci and isolate DNA for genotyping. In order to maximize the number of (different) strains identified, surveys were performed in neighborhoods from which clinical cases had been reported, but diversity was maintained by sampling a maximum of three humans per household. Seven new human sub-clinical cases, with at most mild symptoms, were thus identified, out of 513 individuals screened. This confirms that (i) active case finding is necessary to avoid further increases in advanced clinical VL, and (ii) as reported for other regions including southern European countries, asymptomatic carriers contribute to the transmission cycle. Even more worrying, the incidence among dogs, especially over 1.5 years of age, was over 20 %. No doubt this contributes to the relatively high infection rate in sand flies that we observed in the same region (2.6 % of females - double that of Kutaisi and similar to Tbilisi [8].

Trapping of *Phlebotomus* sand flies allowed the identification of potential vector species that actually carry *Leishmania* parasites, and the isolation of DNA from those protozoans. The most prevalent sand fly species in both Kakhetian districts was *P. kandelakii*, which we previously found highly abundant in western Tbilisi [8], but not in Kutaisi. *Phlebotomus balcanicus* was also found in both districts, which was also observed in all Tbilisi districts and was the most prevalent species in Kutaisi. Finally, we observed some *P. sergenti* in the Kvareli District but not in Sagarejo although we had previously found it in both Tbilisi and Kutaisi [8]. These findings show that a number of *Phlebotomus* species are distributed through most of the country, whereas some appear to have a more local distribution (e.g. *P. wenyoni* in western Tbilisi [8]). Combining the previous Kutaisi/Tbilisi study [8] with the current survey, only *P. kandelakii* (*n* = 17) and *P. balcanicus* (*n* = 5) have been found to be infected with *Leishmania* parasites anywhere in Georgia. Nevertheless large-scale surveys would be necessary to authoritatively confirm that these two species are the only VL vectors in the country.

Malaria control efforts in eastern Georgia in the 1960s included massive spraying campaigns with the insecticide dichlorodiphenyltrichloroethane (DDT) [8], which is believed to have also caused a significant reduction in the sand fly population, as during the next 40 years, until the 1990s, only sporadic VL cases were registered, and only in the extreme eastern part of the country. The existence of active VL foci in border region with Azerbaijan, however, may be facilitating the re-emergence of VL in Kakheti region [9].

We performed the first genotyping of *Leishmania* isolates in the Caucasus region, reporting here ITS-based molecular patterns in *L. donovani* complex samples originating from different geographical areas of eastern Georgia. The distinguishing residues observed in DR 6 and 7 (ITS-2) are entirely unique to the Georgian (and one Azerbaijani) strains reported here, and are not shared by other international isolates currently represented in the GenBank database. Future studies are needed to determine the population structure of *Leishmania* in Georgia in greater detail using additional markers, as well as to explore the prevalence and diversity of major genotypes. The molecular data presented here illustrate that in Tbilisi and other VL foci of Georgia, genetically diverse *Leishmania* subtypes of the *L. donovani* complex are likely responsible for both human and canine leishmaniasis. Our data revealed preliminary insights into the genetic structure of *L. donovani* complex strains currently circulating in Georgia and demonstrate the utility of ITS-based *Leishmania* detection in the resource-limited country of Georgia. An expanded genetic interrogation using additional, higher resolution markers may provide deeper phylogenetic signal and will facilitate greater resolution of the genetic relationships within the *L. donovani* complex in the Caucasus.

## Conclusions

In conclusion, this study confirms that VL remains the most serious vector-borne infection in Georgia, particularly affecting younger children and infants. The high incidence in canines is likely to keep the transmission levels high, as almost all households in rural Georgia keep dogs, as do many in the cities; furthermore, there are many stray dogs that appear to help spread the disease to new areas. In order to break the transmission cycle, vector control would appear to be the most effective option, given the already very high prevalence in domestic and stray dogs, and the absence of affordable, effective treatment for canine leishmaniasis. Although five *Phlebotomus* sand fly species have been identified in the country, to date only two have been found to carry *Leishmania* parasites and it is on these species, particularly, that control measures must focus; further research on their prevalence in other parts of the country will be necessary. Finally, we report the first ITS-based detection of *Leishmania* strains in the Caucasus region and show that these strains are part of the *L. donovani* complex with selected genotypes that are unique to the region. Such data provide further insights into the genetic structure of this pathogen in Eurasia and may inform future comparative studies focused on delineating regional subtypes and any impact on the epidemiology of disease.

## Additional files

Additional file 1: Figure S1. Consensus maximum likelihood (ML) dendogram inferred from concatenated *L. donovani* complex rDNA ITS sequences under the JC best-fit model. (PDF 49 kb) Additional file 2: Figure S2. Partial alignment illustration of major regions of ITS1/2 sequence diversity. (PDF 118 kb) Additional file 3: Full alignment. FASTA file of concatenated *L. donovani* complex ITS sequences included in this study. (FASTA 76 kb)

## Abbreviations

AIC: Akaike Information Criterion
BIC: Bayesian Information Criterion
DR: diversity region
ITS: Internal transcribed spacer
JC: Jukes-Cantor
ML: maximum likelihood
NJ: neighbor joining
NNN: Novy-MacNeal-Nicolle medium
SNP’s: single nucleotide polymorphisms
