# Supplementary figures and images for: Seroepidemiology and molecular diversity of Leishmania donovani complex in Georgia
Source: Parasit Vectors. 2016 May 13;9:279. doi: 10.1186/s13071-016-1558-6 (PMC4866401; doi:10.1186/s13071-016-1558-6)

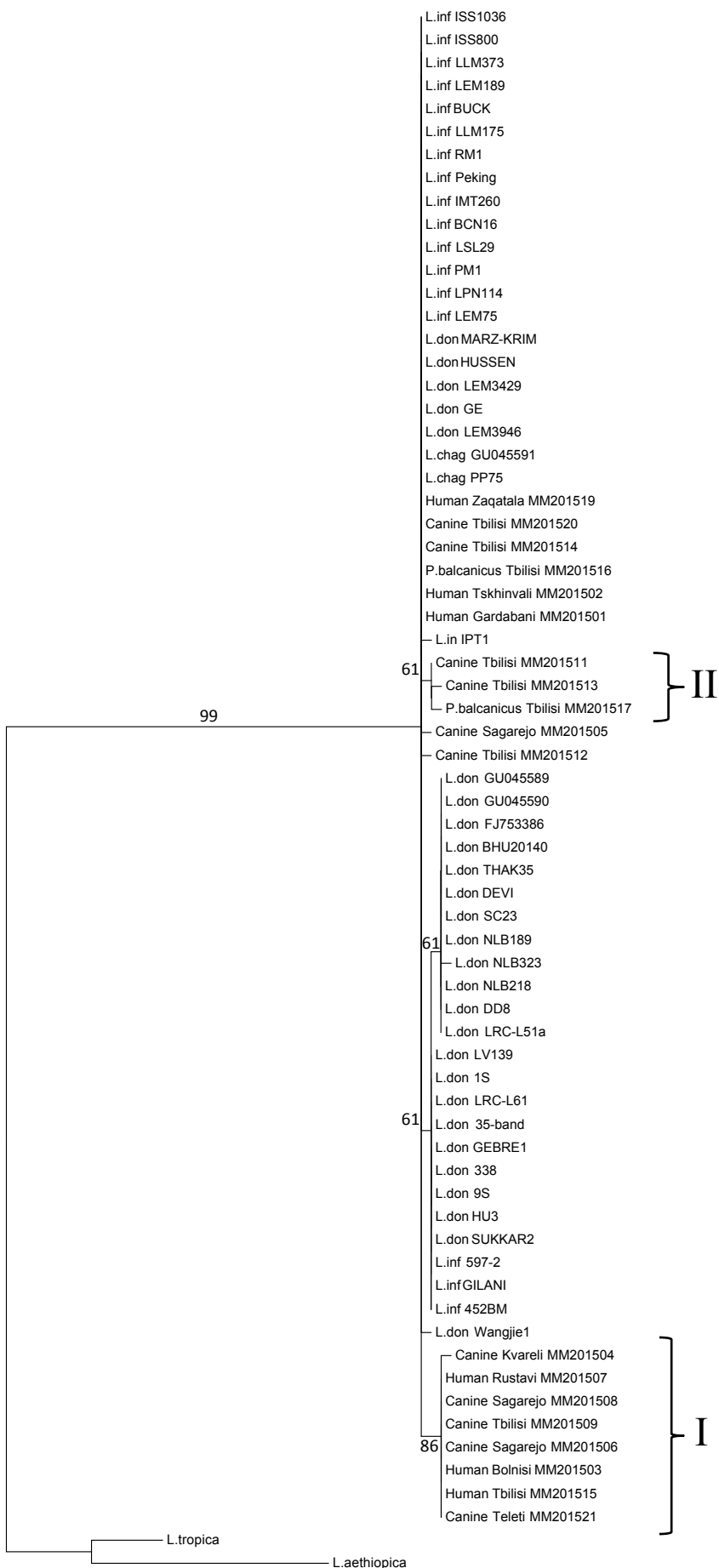

Supplement: Additional file 1: Figure S1. — Consensus maximum likelihood (ML) dendogram inferred from concatenated L. donovani complex rDNA ITS sequences under the JC best-fit model. (PDF 49 kb) [file 13071_2016_1558_MOESM1_ESM.pdf]
