# Supplementary material for: Seroepidemiology and molecular diversity of Leishmania donovani complex in Georgia
Source: Parasit Vectors. 2016 May 13;9:279. doi: 10.1186/s13071-016-1558-6 (PMC4866401; doi:10.1186/s13071-016-1558-6)
Supplement: Additional file 2: Figure S2. — Partial alignment illustration of major regions of ITS1/2 sequence diversity. (PDF 118 kb) [file 13071_2016_1558_MOESM2_ESM.pdf]

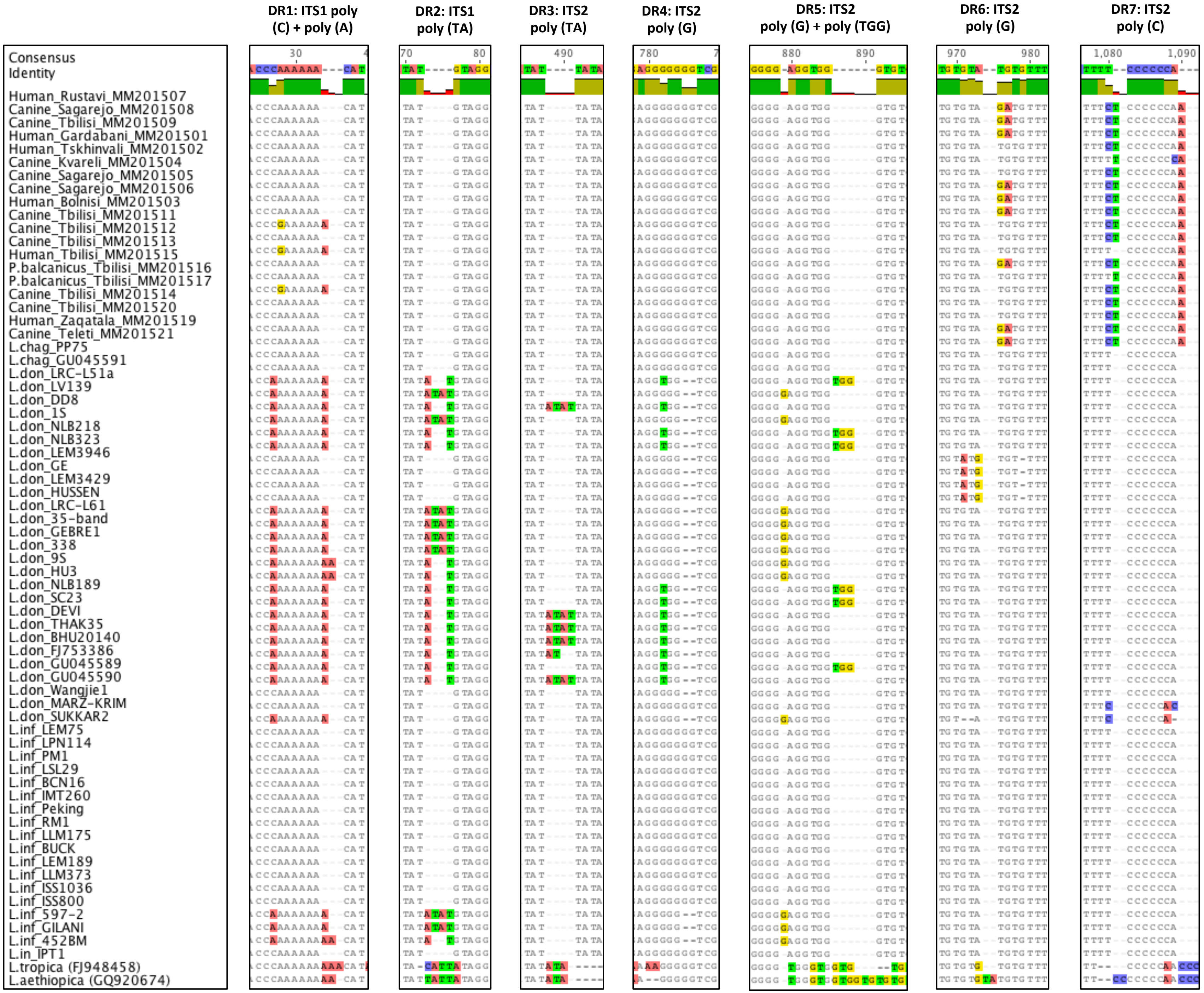
DR1: ITS1 poly (C) + poly (A)
DR2: ITS1 poly (TA)
DR3: ITS2 poly (TA)
DR4: ITS2 poly (G)
DR5: ITS2 poly (G) + poly (TGG)
DR6: ITS2 poly (G)
DR7: ITS2 poly (C)
Consensus
Identity
Human_Rustavi_MM201507
Canine_Sagarejo_MM201508
Canine_Tbilisi_MM201509
Human_Gardabani_MM201501
Human_Tskhinvali_MM201502
Canine_Kvareli_MM201504
Canine_Sagarejo_MM201505
Canine_Sagarejo_MM201506
Human_Bolnisi_MM201503
Canine_Tbilisi_MM201511
Canine_Tbilisi_MM201512
Canine_Tbilisi_MM201513
Human_Tbilisi_MM201515
P.balcanicus_Tbilisi_MM201516
P.balcanicus_Tbilisi_MM201517
Canine_Tbilisi_MM201514
Canine_Tbilisi_MM201520
Human_Zaqatala_MM201519
Canine_Teleti_MM201521
L.chag_PP75
L.chag_GU045591
L.don_LRC-L51a
L.don_LV139
L.don_DD8
L.don_1S
L.don_NLB218
L.don_NLB323
L.don_LEM3946
L.don_GE
L.don_LEM3429
L.don_HUSSEN
L.don_LRC-L61
L.don_35-band
L.don_GEBRE1
L.don_338
L.don_9S
L.don_HU3
L.don_NLB189
L.don_SC23
L.don_DEVI
L.don_THAK35
L.don_BHU20140
L.don_FJ753386
L.don_GU045589
L.don_GU045590
L.don_Wangjie1
L.don_MARZ-KRIM
L.don_SUKKAR2
L.inf_LEM75
L.inf_LPN114
L.inf_PM1
L.inf_LSL29
L.inf_BCN16
L.inf_IMT260
L.inf_Peking
L.inf_RM1
L.inf_LLM175
L.inf_BUCK
L.inf_LEM189
L.inf_LLM373
L.inf_ISS1036
L.inf_ISS800
L.inf_597-2
L.inf_GILANI
L.inf_452BM
L.in_IPT1
L.tropica (FJ948458)
L.aethiopica (GQ920674)
